# Detection of intimate partner aggression through dark personality and moral disengagement

**DOI:** 10.1590/0102-311XEN073523

**Published:** 2023-09-18

**Authors:** Laura Ferreiros, Miguel Clemente

**Affiliations:** 1 Universidade da Coruña, A Coruña, España.

**Keywords:** Intimate Partner Violence, Morality, Personality Assessment, Social Learning, Social Psychology, Violencia de Pareja, Moralidad, Determinación de la Personalidad, Aprendizaje Social, Psicología Social, Violência por Parceiro Íntimo, Moralidade, Determinação da Personalidade, Aprendizado Social, Psicologia Social

## Abstract

Health professionals are often the first to detect abuse. Intimate partner aggression can be approached by studying the personality of the aggressor. From this perspective, dark personality and the use of moral disengagement mechanisms are two key variables. In this study, information was collected from 348 individuals, mostly women, and a questionnaire was applied to determine what their sexual behavior was like with their partner. The *Short Dark Tetrad* (SD4) test was used to identify dark personality and the *Propensity to Moral Disengagement* to identify moral disengagement. Data were collected via Google forms and multivariate decision tree analysis (CHAID growth method) was performed. Results show that men score higher on the variables of dark personality variables and use of moral disengagement mechanisms than on the three variables that, along with sex, increase the presence of dark personality and moral disengagement mechanisms: infidelity, pornography consumption, and maintaining homosexual relationships. Infidelity and pornography consumption are characteristics present in all dimensions of dark personality. Moreover, these three variables are defining characteristics of relationships with high sadism scores. Nursing staff are possibly able to detect the presence of these variables in aggressors and thus initiate a process of victim protection. Therefore, this study proposes that nursing staff should be trained to perform this detection.

## Resumen

Los profesionales de la salud son a menudo los primeros en detectar abusos. La agresión en la pareja íntima se puede abordar a partir del estudio de la personalidad del agresor. Desde esta perspectiva, la personalidad oscura y el uso de mecanismos de desconexión moral son las dos variables principales. Para ello, se recopiló información de 348 personas, casi todas mujeres, y se les aplicó un cuestionario para determinar el comportamiento de la pareja íntima: la prueba *Short Dark Tetrad* (SD4) para identificar la personalidad oscura y la escala *Propensity to Moral Disengagement*. Los datos se recopilaron a través de formularios de Google y se aplicó el análisis estadístico multivariado del árbol de decisión. Los resultados muestran cómo los hombres obtienen puntajes más altos en las variables de personalidad oscura y en el uso de mecanismos de desconexión moral. Además, la infidelidad, el consumo de pornografía, el mantenimiento de las relaciones homosexuales, la infidelidad y el consumo de pornografía se asocian con el logro de puntajes más altos en personalidad oscura y desconexión moral. Además, el mantenimiento de las relaciones homosexuales junto con la infidelidad y el consumo de pornografía son características que definen las relaciones con altas puntuaciones de sadismo cotidiano. Se estima que el equipo de enfermería puede detectar la presencia de estas variables en los agresores y así iniciar un proceso de protección a la víctima. Se propone formar al personal de enfermería para llevar a cabo esta detección.

## Resumo

Os profissionais de saúde são, muitas vezes, os primeiros a detectar abusos. A agressão por parceiro íntimo pode ser abordada a partir do estudo da personalidade do agressor. Nessa perspectiva, a personalidade sombria e o uso de mecanismos de desengajamento moral são as duas principais variáveis. Para isso, foram coletadas informações de 348 pessoas, quase todas mulheres, e aplicou-lhes um questionário para determinar o comportamento do parceiro íntimo: o teste *Short Dark Tetrad* para identificar personalidade sombria e a escala *Propensity to Moral Disengagement*. Os dados foram coletados por meio de formulários do Google e a análise estatística multivariada em árvore de decisão foi aplicada. Os resultados mostram como os homens obtêm escores mais altos nas variáveis de personalidade sombria e no uso de mecanismos de desengajamento moral. Além disso, infidelidade, consumo de pornografia, manutenção de relacionamentos homossexuais, infidelidade e consumo de pornografia estão associados à obtenção de escores mais altos em personalidade sombria e desengajamento moral. Além disso, a manutenção de relacionamentos homossexuais, juntamente com a infidelidade e o consumo de pornografia, são características que definem relacionamentos com altos escores de sadismo cotidiano. Estima-se que a equipe de enfermagem possa detectar a presença dessas variáveis nos agressores e, assim, iniciar um processo de proteção à vítima. Propõe-se que a equipe de enfermagem possa ser treinada para realizar essa detecção.

## Introduction

Intimate partner aggression, also called intimate partner violence, has become a public health problem that includes physical, sexual, and psychological violence and the combination of all of them. Women who are victims of domestic violence suffer more from health problems and therefore need to seek medical services more frequently. Nursing and midwives are often a key player in detecting this type of abuse in both women and children ^1^.

Some cases of intimate partner aggression are detected directly in routine consultations and many others in consultations with women with some risk factor. Moreover, many health institutions implement models to detect risk factors or indicators of violence against women in their emergency services, primary care consultations, or community centers ^1^.

Thus, emergency nurses play a key role in detecting women who are victims of this type of violence, as they are the first to have contact with victims of violence. Therefore, they need to know how to act precisely at that moment, since women are in a very vulnerable position and are most receptive to receiving help. The care of these women in medical services must be perceived empathetically and, above all, away from any judgment about their situation ^2^.

At the same time, some psychological aspects of victims can be predictors of domestic violence, such as low self-esteem, repression, or the fear of showing themselves and answering certain questions ^3^.

Nurses are often the first to detect abuse. Intimate partner aggression can also be approached from the study of the personality of the aggressor. From this perspective, dark personality (Machiavellianism, subclinical narcissism, subclinical psychopathy, and everyday sadism) and the use of moral disengagement mechanisms are two key variables. This study aims to assess which variables of both concepts are related to intimate partner aggression, so that nursing staff can detect possible aggressors and initiate a process of victim protection.

### Dark personality and moral disengagement

Dark personality includes several traits, such as Machiavellianism, subclinical narcissism, subclinical psychopathy, everyday sadism, antisocial behavior, lying, propensity for revenge, among other variables ^4^, which constitute a behavior pattern characterized as “dark”. The dark tetrad of personality involves four components that share certain characteristics and are related to each other but can also be studied separately to understand how they influence the individuals who have them. The four constructs define a dark and socially destructive character, with behavioral tendencies such as grandiosity, emotional coldness, manipulation of others, and aggressiveness.

Some studies try to show whether the components of dark personality vary in a coordinated way or not. A meta-analytical review by Kjaervik & Bushman ^5^ states that subclinical narcissism is the trait that is most closely related to intimate partner aggression, and is even greater in the case of provocation.

A study by Plouffe et al. ^6^ showed that being a woman, as well as the severity of intimate partner aggression and subclinical psychopathy, significantly increased the likelihood of perpetrating severe domestic violence.

Bandura et al. ^7^ proposed, within the framework of social cognitive theory, the concept of moral disengagement as a buffer between individuals’ moral principles and their actual behavior. This concept refers to a psychological schema by which moral authorizations can be disconnected from behaviors that would be harmful, turning harmful acts into acceptable attitudes and allowing immoral and antisocial behaviors to take place. In 1996, they proposed eight mechanisms to measure moral disengagement: moral justification, euphemistic labeling, advantageous comparison, displacement of responsibility, diffusion of responsibility, distortion of consequences, dehumanization, and attribution of blame. Individuals with a high score on dark personality traits use more moral disengagement mechanisms to distress their victims ^8^ ^,^ ^9^.

Both dark personality and moral disengagement mechanisms are related to and can even be predictors of domestic violence. All dark personality traits are related to domestic violence, but subclinical psychopathy is the trait most related to verbal, physical, and sexual violence.

This study aims to provide nursing staff with knowledge to detect intimate partner aggression based on the personality of the possible aggressors. Measuring the presence of the dimensions of dark personality and moral disengagement in both members of the couple, especially in the alleged aggressor, would be most appropriate, but if the tools can not be applied, nursing staff should be able to identify their signs to a large extent.

Therefore, we formulated the following hypotheses:

(a) H1: the greater the presence of dark personality variables and the use of moral disengagement mechanisms, the greater the likelihood of perpetrating intimate partner aggression;

(b) H2: the most determining variables when suspecting that a woman may is suffering from intimate partner aggression are frequent infidelities, use of pornography, and non-consensual sexual activities;

(c) H3: in homosexual relationships, the possibility of aggression against the partner is higher than in heterosexual relationships.

## Methods

The study design was descriptive and correlational. However, in the final part of the research, the variable collected in the ad hoc questionnaire were considered causal and those collected by the dark personality and moral disengagement questionnaires were dependent.

### Participants

Information was collected from 348 individuals, of whom 21.3% were men and 78.7% women. They were all young adults, aged 18 to 25 years (mean = 22.14, SD = 1.53). In total, 59.61% were university students, 20.59% had already finished their university studies, and 19.8% had non-university studies. Of the participants, 75.9% had already had a romantic partner at some point, and 24.1% had not. Individuals who had never had a partner were eliminated, totaling 264 participants in the final sample. The profile of the sample was therefore university students, mostly women, young, and with a partner. Data were collected by a questionnaire on Google Forms (https://workspace.google.com) and snowball sampling was conducted using social networks, making the sampling non-probabilistic.

### Data collection

A Google Forms questionnaire was created. The team of collaborators included students from the subjects taught by the researchers (three collaborators). They were instructed about the study and their role was to disseminate the questionnaire on their social media. They had a specific temporary period to collect information: May to June 2021.

The Google Forms questionnaire included the following sections: explanation of the research and computerized consent request, including the researchers’ contact details in case any participant had any questions; sociodemographic data; identification of intimate partner behavior; dark personality scale; and moral disengagement scale. Each tool is described below, except for the one that collected participants’ sociodemographic data.

(a) Identification of intimate partner behavior: this ad hoc tool was created for this study. It included questions about participants’ sexual orientation; their current relationship; length of relationship with their current partner; whether the relationship was monogamous or not; whether the relationship was heterosexual, homosexual, or other; length of their longest relationship; number of partners throughout their life; whether or not they had ever been unfaithful to their partner or relapsed into infidelity; whether they consumed pornography; whether their partner was aware of this consumption; whether they had ever been forced to perform any sexual practice by their partner; and whether they had ever stopped performing any sexual practice to satisfy their partner. The questions were extracted from reading others studies ^10^ ^,^ ^11^ ^,^ ^12^.

Control questions were placed between the questions of the questionnaire to confirm that participants were paying attention to both the questions and their answers (for example, “This is a control question, check option 4”). All questions were mandatory, so participants had to answer all of them.

(b) SD4 scale: the *Short Dark Tetrad* scale was used to measure dark personality traits: Machiavellianism, subclinical narcissism, subclinical psychopathy, and everyday sadism. This questionnaire was created by Paulhus et al. ^4^ and consists of 28 items rated on a 5-point Likert scale ranging from 1 (strongly disagree) to 5 (strongly agree). Each subscale has seven items. An example item is: “It’s not wise to let people know your secrets” (item 1, Machiavellianism subscale). The reliability of each subscale was determined with Cronbach’s alpha index, which was acceptable in all of them: 0.62 for Machiavellianism, 0.76 for subclinical narcissism, 0.75 for subclinical psychopathy, and 0.73 for everyday sadism.

(c) PMD scale: the *Propensity to Morally Disengage* (PMD) scale ^11^ was used to obtain information about moral disengagement mechanisms. It has 24 items, three items for each moral disengagement mechanism (moral justification, euphemistic labeling, advantageous comparison, displacement of responsibility, diffusion of responsibility, distortion of consequences, dehumanization, and attribution of blame). Items are rated on a 7-point Likert scale ranging from 1 (strongly disagree) to 7 (strongly agree). Each subscale has seven items. An example item is: “It is okay to spread rumors to defend those you care about” (item 1, moral justification subscale). The reliability of the subscales was determined with Cronbach’s alpha index: the average of the reliability coefficients was 0.55, which is somewhat low due to the size of the sample and its homogeneity.

### Data analysis

Once data were collected, they were downloaded into Excel (https://products.office.com/) and prepared for import into SPSS version 26 (https://www.ibm.com/). After cleaning the data, the following statistical analyses were performed: estimation of the percentages of appearance of each qualitative variable (performed specifically with the variables of the scales for collecting sociodemographic data and information on intimate partner behavior); estimation of central tendency and dispersion values for the qualitative variables (applied to the SD4 and PMD scales); estimation of the reliability of the two scales using Cronbach’s alpha index; and application of the classification tree technique, considering each dimension of the SD4 and PMD scales as a dependent variable and all variables included in the intimate partner behavior questionnaire as independent variables. Decision tree analysis helps identify group characteristics, assess the relationships between independent and dependent variables, and presents this information in a non-technical way.

### Ethical considerations

Before starting the questionnaire, participants read a brief description of the study and, after completing it, gave their informed consent.

This study was approved by the Research Ethics Committee of the Criminology and Legal Psychology Research Group, Coruna University (title: *Intimate Partner Aggression and Dark Personality*, ID: 2021/52). It complies with the ethical criteria of the Helsinki protocol and the American Psychological Association. 

This study was not funded by any public, commercial, or non-profit funding agency.

## Results

### Intimate partner behavior

The first questionnaire included questions on the participants’ sexual identity and showed the following results:

(a) Sexual orientation: 81.1% were heterosexual, 13.7% bisexual, 4.7% homosexual, and 0.3% selected other alternatives; 

(b) In total, 54.1% had a partner at the time of the study, while 45.9% did not;

(c) Regarding the length of relationship with their current partner, most participants (29%) had been with their current partner for less than one year, followed by more than four years (24.2%), less than two years (18.8%), less than four years (15.6%), and less than three years (12.4%);

(d) The type of relationship of most participants was monogamous (95.2%), and only in 4.8% of cases was it not;

(e) In most cases, the relationship was heterosexual and, to a much lesser extent, homosexual (94.6% versus 5.4%, respectively);

(f) Regarding the length of their longest relationship, most participants answered less than one year (35.8%), followed by less than two years (30.8%) and more than four years (10%). The longest relationships were always monogamous (96.6%) and heterosexual (94.2%);

(g) Regarding the number of partners throughout participants’ life, the most common answer was two or three (54.9%), followed by only one (34%);

(h) In 24.8% of cases, participants had already been unfaithful to their partner (thus, this did not occur in 75.2%). Moreover, 40.8% of participants had been unfaithful to the same partner on more than one occasion;

(i) Of the participants, 47.4% consumed pornography, and in 78.6% of these cases, the partner was aware of this consumption. Moreover, in 23.7% of cases, consumption was joint;

(j) In total, 7.5% of participants had already been forced by their partner to perform some kind of sexual practice against their will;

(k) Finally, 48.4% of participants had ever put aside their sexual preferences to satisfy their partner, while 51.6% had not.

Based on these data, we were able to determine the profile of the young adult participants. They are usually heterosexual, with almost equal probability of having a partner or not, and consider it normal to have been with their partner for less than one year. Their relationships are generally monogamous and heterosexual and usually last less than one year. Relationships that last longer are generally heterosexual and monogamous. They have usually had two or three partners throughout their lives and are generally faithful to their partners. However, in the same proportion, they have already been unfaithful to the same partner more than once. In half of the cases, they consume pornography, and in a quarter of the cases, consumption is joint. Usually, the couple is aware of pornography consumption. Although most young adults had not been forced by their partner to perform sexual practices against their will, 7.5% experienced this. Finally, almost half of the participants had stopped performing sexual practices to satisfy their partner.

We measured four dark personality variables and eight moral disengagement variables. Table 1 presents their descriptive statistics. The mean scores are close to the midpoints of the scales (3 for the SD4 scale and 4 for the PMD), except for everyday sadism, advantageous comparison, and attribution of blame, where the scores are significantly lower. Certainly, this sample has scores similar to the normal population. We performed a t-test to determine the differences between men and women, and all results were significant.

**Table 1:** Descriptive statistics for quantitative variables (*Short Dark Tetrad* scale and *Propensity to Morally Disengage* scale).

Variables	Minimum	Maximum	Mean	t-test differences between men-women	SD
Machiavellianism	1.14	4.43	2.638	3.580 *	0.661
Narcissism	1.00	4.86	2.461	5.049 *	0.722
Psychopathy	1.00	4.29	1.693	4.033 *	0.617
Sadism	1.00	4.71	1.954	3.887 *	0.709
Moral justification	1.00	6.00	2.664	5.001 *	1.231
Euphemistic labeling	1.00	7.00	2.291	3.434 *	1.039
Advantageous comparison	1.00	5.33	1.899	2.998 *	0.902
Displacement of responsability	1.00	6.00	2.188	4.093 *	0.973
Diffusion of responsability	1.00	5.67	2.006	4.667 *	0.944
Distortion of consequences	1.00	5.33	1.996	3.112 *	0.862
Dehumanization	1.00	6.33	2.461	4.002 *	1.201
Attribution of blame	1.00	4.00	1.553	2.999 *	0.6127

SD: standard deviation.

* p < 0.01.

Dark personality and moral disengagement variables were dependent and sexual identification variables were independent. We used the multivariate decision tree technique and, at all times, the CHAID growth method. This study omitted the results in case the only intervening variable in the nodes was sex, since the literature consistently shows that men score higher than women in all the variables used. Therefore, we obtained no relevant information. The following section presents the results for dark personality.

### Dark personality

#### Machiavellianism 

**Figure 1:**
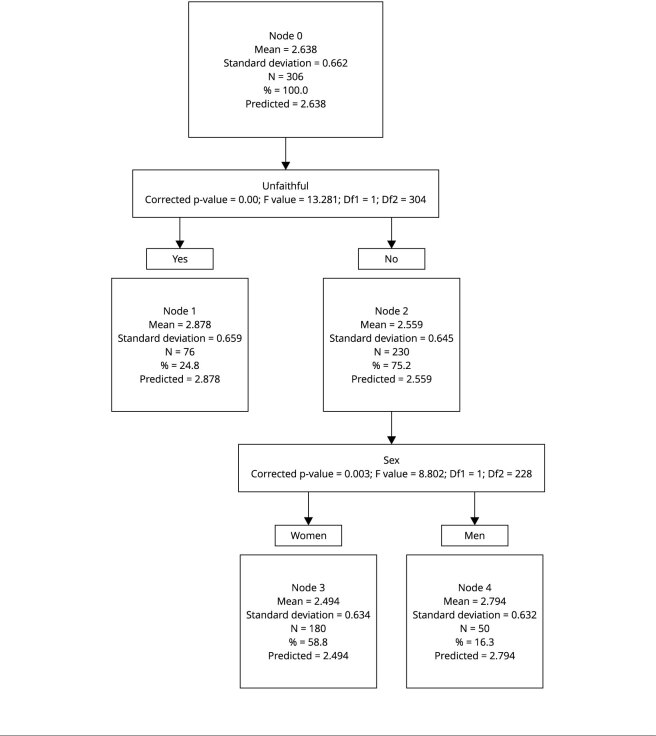
Classification tree technique for Machiavellianism.

In this case, the maximum depth of the tree was 3 (Figure 1). The minimum number of cases in a child node was 100 and in a parent node 50. The number of nodes was five, of which two were terminal. The depth of the tree was 2, and we obtained the following gains for the nodes: node 1, 24.8%, mean = 2.877; node 2,75,2%, mean = 2,589; node 3, 58.8%; mean = 2.493; node 4, 16.3%, mean = 2.794. The risk estimate was 0.406, with a standard error of 0.028. This tree shows that the level of Machiavellianism is higher in individuals who were unfaithful compared with faithful participants. Moreover, among faithful individuals, Machiavellianism is higher in men. In general, dark personality and moral disengagement variables are more present in men than in women.

For subclinical narcissism and subclinical psychopathy, sex was the only significant classificatory variable. This study presents the tree for everyday sadism below. Given the large number of figures we could include, we decided to use Figure 1 as an example to comment on the results. The authors will provide the remaining figures upon request.

#### Everyday sadism

In this case, the maximum depth of the tree was 3. The minimum number of cases in a child node was 100 and in a parent node 50. The number of nodes was seven, of which four were terminal. The depth of the tree was 3, and we obtained the following gains for the nodes: node 2, 21.9%, mean = 2.539; node 4, 29.4%, mean = 1.906; node 6, 18%, mean = 1.836; node 5, 30.7%, mean = 1.653. The risk estimate was 0.396, with a standard error of 0.036. This tree shows that the level of everyday sadism is higher in women in homosexual relationships. Moreover, among women in heterosexual relationships, everyday sadism is higher in those who consumed pornography.

### Moral disengagement

#### Moral justification

This variable showed a classificatory tree with significant explanatory variables of this mechanism. In this case, the maximum depth of the tree was 3. The minimum number of cases in a child node was 100 and in a parent node 50. The number of nodes was five, of which three were terminal. The depth of the tree was 2, and we obtained the following gains for the nodes: node 1, 24.8%, mean = 3.083; node 4, 29.34%, mean = 2.827; node 3, 40.5%, mean = 2.269. The risk estimate was 1.396, with 0.110 standard error. This tree shows that the mechanism of moral justification is present to a greater extent in unfaithful people. Moreover, among faithful individuals, moral disengagement is more present in the case of pornography consumption.

#### Euphemistic labeling

In this case, the maximum depth of the tree was 3. The minimum number of cases in a child node was 100 and in a parent node 50. The number of nodes was five, of which three were terminal. The depth of the tree was 2, and we obtained the following gains for the nodes: node 1, 24.8%, mean = 3.083; node 4, 29.34%, mean = 2.827; node 3, 40.5%, mean = 2.269. The risk estimate was 1.396, with a standard error of 0.110. This tree shows that euphemistic labeling is more present in unfaithful individuals. Moreover, among faithful individuals, this mechanism is used more in the case of pornography consumption.

#### Advantageous comparison

In this case, the maximum depth of the tree was 3. The minimum number of cases in a child node was 100 and in a parent node 50. The number of nodes was five, of which three were terminal. The depth of the tree was 2, and we obtained the following gains for the nodes: node 1, 24.8%, mean = 2.232; node 4, 34.6%, mean = 1.924; node 3, 40.5%, mean = 1.672. The risk estimate was 0.763, with a 0.080 standard error. The risk estimate was 1.396, with a 0.110 standard error. This tree shows that this mechanism is more present in unfaithful individuals. Among faithful individuals, advantageous comparison is used more in the case of pornography consumption.

#### Displacement of responsibility

In this case, the maximum depth of the tree was 3. The minimum number of cases in a child node was 100 and in a parent node 50. The number of nodes was three, of which two were terminal. The depth of the tree was 1, and we obtained the following gains for the nodes: node 1, 24.8%, mean = 2.434; node 2, 75.2%, mean = 2.107. The risk estimate was 0.925 with a 0.090 standard error. This tree shows that this mechanism is used more by unfaithful individuals.

#### Diffusion of responsibility

In this case, the maximum depth of the tree was 3. The minimum number of cases in a child node was 100 and in a parent node 50. The number of nodes was of seven, of which four were terminal. The depth of the tree was 3, and we obtained the following gains for the nodes: node 2, 21.9%, mean = 2.318; node 3, 19.3%, mean = 2.158; node 6, 21.9%, mean = 2.019; node 5, 38.9%, mean = 1.734. The risk estimate was 0.836, with a standard error of 0.079. This tree shows that this mechanism is more present in men than in women. Moreover, it is used more by unfaithful women compared with faithful women. On the other hand, among faithful women, diffusion of responsibility is more present in faithful women who consume pornography.

#### Distortion of consequences

In this case, the maximum depth of the tree was 3. The minimum number of cases in a child node was 100 and in a parent node 50. The number of nodes was three, of which two were terminal. The depth of the tree was 1, and we obtained the following gains for the nodes: node 1, 24.8%, mean = 2.346; node 2, 75.2%, mean = 1.881. The risk estimate was 0.701, with a 0.069 standard error. This tree shows that this mechanism is used more by unfaithful individuals than by faithful individuals.

Attribution of blame: In this case, the maximum depth of the tree was 3. The minimum number of cases in a child node was 100 and in a parent node 50. The number of nodes was seven, of which four were terminal. The depth of the tree was 3, and we obtained the following gains for the nodes: node 2, 21.9%, mean = 1.925; node 3, 19.3%, mean = 1.599; node 6, 21.9%, mean = 1.507; node 5, 36.9%, mean = 1.336. The risk estimate was 0.326, with a standard error of 0.032. This tree shows that this mechanism is used more by men than by women. Moreover, attribution of blame is more present in unfaithful women compared with faithful women. On the other hand, among faithful participants, this mechanism is used more by individuals who consume pornography.

This study does not present the information resulting from the consideration of dehumanization as a dependent variable, since it only provided valid comparisons based on the sex variable, thus showing that the mechanism is used more by men than by women.

## Discussion

The results of this study confirm the proposed hypotheses. Moreover, these results are consistent with previous studies on the topic. Our study is in line with almost all studies that show that dark personality traits are more present in men than in women ^12^ ^,^ ^13^. Studies show that dark personality traits increase psychological violence in romantic partners. However, this increase is not the same for all personality traits. Some authors state that women use violence against their partners on a regular basis ^13^. Green et al. ^14^ pointed that, at least regarding subclinical narcissism, research samples are generally men and, therefore, they score higher in this trait than women.

The presence of dark personality traits is directly related to having more than one sexual partner, preferring short-term relationships, and living an unrestricted sociosexuality ^15^. Considering that partners of individuals with dark personality traits are also attracted to other people, the variety of partners they may have favor short-term relationships ^9^.

Both short-term relationships and dark personality traits are more associated with men than with women ^9^. This type of relationship is related to infidelity, since it promotes the search for relationships outside the couple to satisfy desires, although it is not related to lack of self-control.

Experiencing unrestricted sociosexuality is especially related to subclinical psychopathy, which facilitates the possibility of finding a partner ^8^. Sometimes these individuals are even willing to lower their expectations of a person in order to have more options for a partner ^16^. On the other hand, women who score high on subclinical narcissism and Machiavellianism maintain expectations of their potential partners, which facilitates possible infidelity ^17^.

### Limitations

This study has limitations. The sample, although considerably large (348 individuals), should be larger in future studies. Similarly, future studies should try to interview both members of the couple in the case of participants who are currently in a relationship. Moreover, our sample was not random, but incidental. Future studies should also broaden the age range of the population, considering not only young adults, which will allow increasing the reliability data of the questionnaires applied. On the other hand, this study is useful to create a profile of toxic individuals who are more likely to be aggressors towards their partners, making them victims. Nursing staff are usually the first contact for victims of abuse and therefore can advise them. The detection of the characteristics summarized at the beginning of the conclusions can stop this process, allowing the action of professionals specialized in this problem.

## Conclusion

The results of this study support the following facts, confirming the three hypotheses raised:

(a) Men score higher than women on all dark personality variables and on the use of all moral disengagement mechanisms;

(b) The three variables that, along with sex, increase the presence of dark personality traits or the use of moral disengagement mechanisms are infidelity, pornography consumption, and maintaining homosexual relationships;

(c) Infidelity is the most present characteristic in all the dimensions of dark personality (which includes both the dimensions of dark personality and moral disengagement mechanisms). It is only absent in subclinical narcissism, subclinical psychopathy, and dehumanization;

(d) Pornography consumption is another characteristic associated with dark personality, especially among individuals scoring high on moral justification, euphemistic labeling, and advantageous comparison;

(e) Maintaining homosexual relationships, along with infidelity and pornography consumption, define relationships that score high on everyday sadism.

These findings are in general agreement with other authors, but the advantage of this study is that it analyzed dark personality and moral disengagement in relationships with intimate partners ^11^. High scores on dark personality and moral disengagement produced a negative interaction with others, characterized in the case of couples by high levels of jealousy, infidelity, and violence.

Health professionals, and especially nursing staff, need to be prepared to care for women who are victims of intimate partner aggression. Therefore, educating them on intimate partner aggression is necessary, so that they can identify it and respond to the abuse suffered by women. Nurses and midwives, the professionals who may be most in contact with women, should be continuously informed about what to do in case of abuse or violence. Knowing how to act in these situations is important, identifying possible cases of abuse and informing institutions when necessary so that they can intervene in the fastest and most effective way ^1^.

Several studies show that health professionals, especially nurses due to their role in medical examinations, who have received training and education on intimate partner aggression are better able to detect this violence than professionals who have not received it ^11^.

Therefore, nursing academic curricula should include different ways to correctly identify signs of violence in the victim, criteria that also avoid errors based on lack of knowledge or prejudice about the economic status, ethnicity, or nationality of both the victim and the aggressor ^18^ ^,^ ^19^. We also suggest including knowledge related to dark personality and moral disengagement.
